# CryoEM structure of the antibacterial target PBP1b at 3.3 Å resolution

**DOI:** 10.1038/s41467-021-23063-6

**Published:** 2021-05-13

**Authors:** Nathanael A. Caveney, Sean D. Workman, Rui Yan, Claire E. Atkinson, Zhiheng Yu, Natalie C. J. Strynadka

**Affiliations:** 1 Department of Biochemistry and Molecular Biology, University of British Columbia, Vancouver, BC Canada; 2 The Centre for Blood Research, University of British Columbia, Vancouver, BC Canada; 3 CryoEM Shared Resources, Howard Hughes Medical Institute, Ashburn, VA USA; 4 HRMEM Facility, University of British Columbia, Vancouver, BC Canada

**Keywords:** Cryoelectron microscopy, Cryoelectron microscopy

## Abstract

The pathway for the biosynthesis of the bacterial cell wall is one of the most prolific antibiotic targets, exemplified by the widespread use of β-lactam antibiotics. Despite this, our structural understanding of class A penicillin binding proteins, which perform the last two steps in this pathway, is incomplete due to the inherent difficulty in their crystallization and the complexity of their substrates. Here, we determine the near atomic resolution structure of the 83 kDa class A PBP from *Escherichia coli*, PBP1b, using cryogenic electron microscopy and a styrene maleic acid anhydride membrane mimetic. PBP1b, in its apo form, is seen to exhibit a distinct conformation in comparison to Moenomycin-bound crystal structures. The work herein paves the way for the use of cryoEM in structure-guided antibiotic development for this notoriously difficult to crystalize class of proteins and their complex substrates.

## Abstract (web summary)

Our structural understanding of class A penicillin binding proteins is incomplete due to the difficulty in their crystallization and the complexity of their substrates. Here, authors determine the structure of the 83 kDa class A PBP from *Escherichia coli*, PBP1b, using cryogenic electron microscopy and a styrene maleic acid anhydride membrane mimetic.

## Introduction

The bacterial cell wall and the enzymes that provide for its biosynthesis have long been targeted clinically, as exemplified by the widespread use of β-lactam antibiotics, which inhibit final crosslinking steps in this pathway^1^. The popularity of cell wall biosynthesis as an attractive set of antibiotic targets is largely a factor of the unique to bacteria nature of several of the enzymes involved and the overall essentiality of the also unique to bacteria cell wall ultrastructure in viability, infection and pathogenesis. This cell wall is composed of an extensive crosslinked mesh composed of peptidoglycan (PG)—polymerized strands of alternating β-1,4-linked *N*-acetyl-glucosamine-*N*-acetyl-muramic acid covalently linked via short peptide cross bridges attached at the C3-OH of the muramic acid^1^.

PG is synthesized using a multistage pathway involving: (1) production of soluble precursors in the bacterial cytosol; (2) precursor assembly onto lipid carriers at the inner leaflet of the cytoplasmic membrane; (3) flipping of the lipid-linked precursors to the outer leaflet of the cytoplasmic membrane; (4) polymerization of the lipid-linked precursors into PG strands; (5) crosslinking of these polymerized PG strands into the existing PG sacculus. The last two steps in this pathway occur through the activity of penicillin-binding proteins (PBPs)^1,2^. PBPs are split into two major classes, class A and B PBPs. Class A PBPs perform both a glycosyltransferase activity (GTase), which polymerizes the lipid activated precursor molecules into a growing PG strand (Supplementary Fig. 1), and a d,d-transpeptidase activity, which catalyses the cleavage of a d-Ala^4^-d-Ala^5^ peptide bond of the acyl donor and subsequently crosslinks the d-Ala^4^ carbonyl to the primary amine of a diaminopimelic acid (DAP) residue on the acceptor (in Gram negatives such as *Escherichia coli*), resulting in a d-Ala^4^_donor_-DAP^3^_acceptor_ crosslink^3^. Monofunctional class B PBPs perform only the latter activity^2,3^. Inhibition of either of these crucial, PBP-mediated, final steps in PG biosynthesis results in destabilization of the cell wall and ultimately cell death^4^. The d,d-transpeptidase activity of both classes can be irreversibly acylated and inhibited by β-lactam antibiotics, which act as a substrate mimetic for the donor strand peptide^3^. The GTase domain can be inhibited by moenomycin, a natural product antibiotic produced by *Streptomyces* soil bacteria; commonly used as a growth promoter in animal feed, the C24 lipidated moenomycin is untenable as an antibiotic in humans due to poor pharmacokinetic properties^3^. The polymerizing GTase domain of class A PBPs consists of a membrane inserted variation on a lysozyme-like donor site, putatively accommodating four sugars and the pyrophosphate lipid of the growing PG strand^3^. A lipid II disaccharide unit binds at the acceptor site, with an intervening strictly conserved Glu providing the catalytic base for the β-1–4 covalent attachment of anomeric C1 of donor and 3′-OH of acceptor^3^ with concomitant loss of C55 pyrophosphate leaving group from the donor. The processive polymerization of the growing donor strand relies on a dynamic membrane-embedded “jaw” domain that putatively changes conformation to allow new product in each round to slide into the donor site^5^. The dynamic motion of the GT domain has indeed also likely been a contributing cause of thwarted high-resolution crystallographic efforts on these bifunctional PBP enzymes despite significant efforts globally. Structures to date have relied on the presence of the moenomycin A (MoeA)^3,6–8^, defining the latter as a donor site substrate analogue that likely helps promote stability of the dynamic GT domain, although with typically still large areas of disorder and thus continuing questions about critical active site features. The ability to capture more complex and typically heterogeneous physiological PG strand substrates for either the TPase or GTase domains has also been a thus far intractable hurdle for crystallographic analysis and again leaving many unanswered questions including acceptor strand binding in the TPase domain, donor binding in the GTase domain, and the presumed intervening path for growing substrate between the two distinct catalytic centres lying >20 Å apart.

Here, we determine the near-atomic resolution structure of the 83 kDa class A PBP from *E. coli*, PBP1b, using cryogenic electron microscopy (cryoEM) and a membrane mimetic. PBP1b, in its apo form, is seen to exhibit a distinct conformation in comparison to moenomycin-bound crystal structures. The work herein paves way for the use of cryoEM in structure-guided antibiotic development for this notoriously difficult to crystalize class of proteins and their complex substrates.

## Results and discussion

### Structure of apo *E. coli* PBP1b

**Fig. 1:**
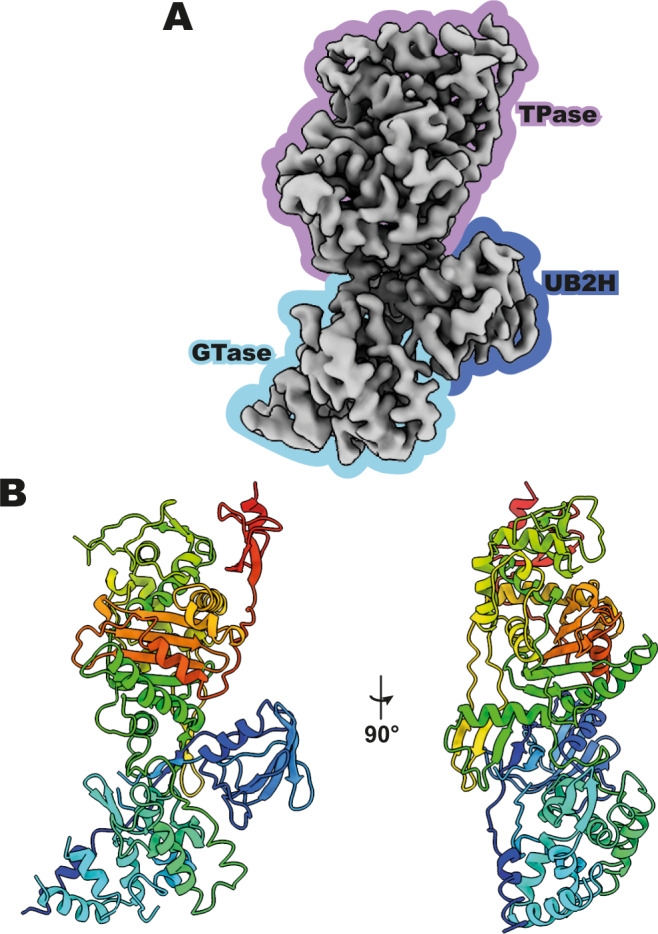
CryoEM structure of apo *E. coli* PBP1b. **A** Reconstruction of apo PBP1b at 3.28 Å resolution with GTase (glycosyltransferase), TPase (transpeptidase) and UB2H (putative activator binding) domains labelled. **B** Refined structure of apo PBP1b in ribbon representation and rainbow colouring from N to C terminus (blue to red).

Detergent-extracted *E. coli* PBP1b (residues 58–804, lacking N- and C-terminal disordered regions)^3^ and PBP1a were reconstituted into polymeric styrene maleic anhydride (SMA), a detergent-free system for imaging via cryoEM^9^. Both PBP1a and PBP1b (Supplementary Fig. 2) exhibited glycerol gradient centrifugation chromatograms and the ability to bind cognate regulators that suggested the enzymes were readily stabilized in SMA, with comparable behaviour to that in detergent micelles such as *N*-dodecyl-d-maltopyranoside (DDM). For the purposes of this manuscript, we focus on the atomic details of the unexplored apo state of *E. coli* PBP1b, although we include initial *E. coli* PBP1a data to show that this methodology is likely applicable to a range of bifunctional PBPs. Initially, the SMA system was used in an attempt to capture the LpoB and LpoA activators, which, in our hands, did not seem to stably bind PBP1b and PBP1a in detergents. In addition, optimization of PBP1b and PBP1a vitrification with sufficient particle density was achieved more readily and at much lower concentrations (0.1–0.15 mg/mL for both PBP1b and PBP1a) in the SMA, as vitrification in detergents can often be challenging and require significantly more protein. Interestingly, the most well-behaved samples post vitrification contained the addition of the known 13 kDa protein activator of PBP1b transpeptidase activity, LpoB (which we were initially attempting to capture—~*K*_d_ with PBP1b = 0.8 μΜ^10^; see “Methods”), although we believe this protocol should work sufficiently in the absence of lipoprotein activators. However, no evidence of LpoB was subsequently observed in any particle classes during downstream cryoEM data processing, suggesting that the protein had been lost from the complex during cryogenesis. Following complete processing of the PBP1b data, including subtraction by masking of clear features corresponding to the SMA membrane mimetic in initial maps, a cryoEM reconstruction of the apo state of *E. coli* PBP1b was produced (Fig. 1A and Supplementary Fig. 3), showing well-resolved map features for all three domains of PBP1b (Fig. 1B) and an overall resolution of 3.28 Å (Supplementary Table 1 and Supplementary Fig. 3). Both before and after particle subtraction, the transmembrane helix was only observed where it packs against the GTase domain. In all, the modelled regions span residues 82–98, 100–207, 212–236, 267–297, 286–542, and 547–798. The overall tri-partite architecture of PBP1b is as previously observed in MoeA-liganded structures^3,7,8^, with the canonical and well-described C-terminal transpeptidase (TPase) domain, central putative activator binding domain (UB2H domain), and N-terminal membrane-anchored glycosyltransferase (GTase) domain all able to be modelled into the cryoEM reconstruction as a complete structure of the apo state of *E. coli* PBP1b.

### Domain rearrangements of *E. coli* PBP1b

**Fig. 2:**
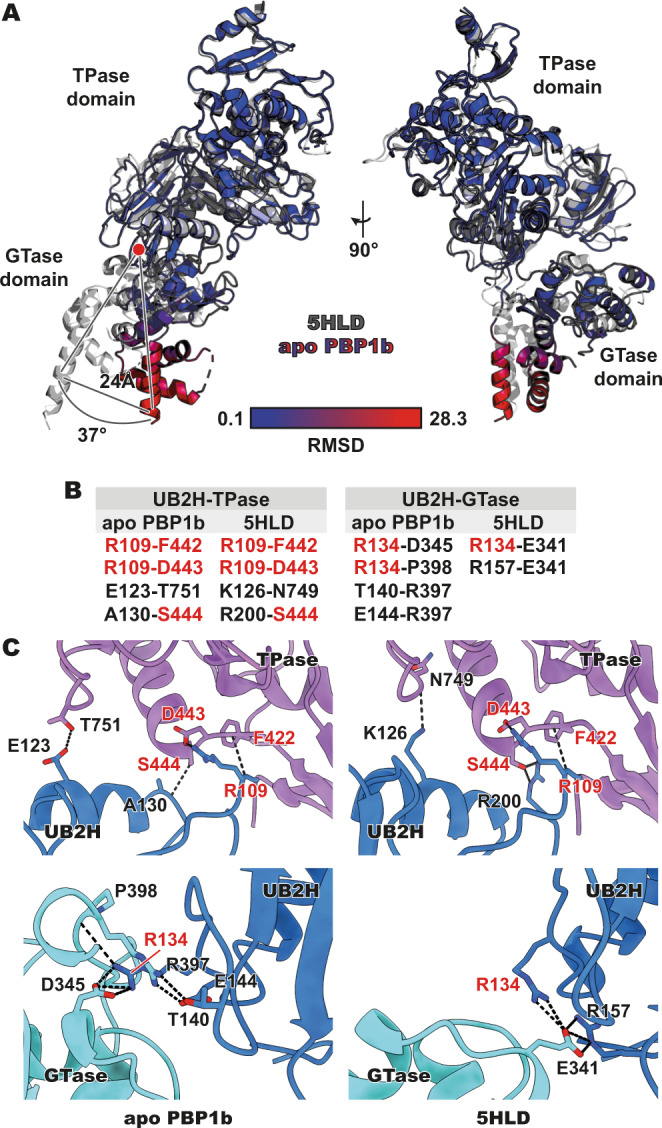
Domain rearrangement and differential inter-domain interfaces. **A** Meropenem- and acyl-CENTA-bound *E. coli* PBP1b (PDB 5HLD) and apo *E. coli* PBP1b superimposed. Liganded PBP1b (PDB 5HLD) is in grey, while residues of apo PBP1b are coloured by RMSD from blue (0.1 Å) to red (28.3 Å), with an average RMSD of 3.2 Å across all modelled residues. There is a striking rotational reorientation of the GTase (glycosyltransferase) domain between the two structures. The GTase domain is seen to rotate 37° and the TM side of the domain is rotated at a distance of 24 Å. **B** The various hydrogen bonds at the domain interfaces (UB2H (putative activator binding)-TPase (transpeptidase) and UB2H-GTase) are listed. Residues and/or residue pairs that play a role in both structures are in red font. **C** The interdomain interfaces (UB2H-TPase and UB2H-GTase) of both apo PBP1b and liganded PBP1b (PDB 5HLD). Residues involved in hydrogen bonding are labelled as in (**B**). Hydrogen bonds are represented by dashed lines. Domains are coloured as in (**B**), with TPase in purple, UB2H in blue, and GTase in cyan.

Despite the expected broad structural conservation of the apo PBP1b in comparison to previously solved MoeA-liganded structures^3,7,8^, significant domain rearrangements are observed between the two states. The TPase domain and the UB2H remain as a near-fixed domain dyad, but with an ~40° relative rotation of the GTase domain with respect to the fixed TPase/UB2H pair (measured by UCSF Chimera^11^; Fig. 2A). These rearrangements are in keeping with those proposed by earlier nuclear magnetic resonance, mutagenesis, and modelling studies of PBP1b fragments in response to binding of the activator protein LpoB^10,12^. In comparison to MoeA-liganded structures of PBP1b, we propose that the observed ~40° relative rotation is likely ligand-induced. In the MoeA-liganded structures, there is a modest displacement of the β-hairpin at residues 315–325, mainly due to an interaction between the terminal amide group of Q318, which is displaced 1.9 Å in comparison to the apo structure. This modest sidechain displacement results in a 2.2 Å displacement of the Cα of Q318, and a displacement for the β-hairpin of 1.6 Å (measured from the Cα of the terminal D321 of the hairpin). As these modest displacements are near to the fulcrum of the pivot between the GTase and TPase domains, they radiate outward into larger displacements, such as a 5.4 Å displacement of the loop (residues 397–411, measured from the Cα of I408) between the GTase and TPase domains and further outward from the fulcrum, displacements of 6.8 Å on the GTase loop residues 489–499 (measured from the Cα of A496). Overall, this results in the relatively significant rotational shift seen for the GTase domain between the two states. Due to the similarity of MoeA to the natural ligands of PBP1b, it is possible that these domain rearrangements may occur naturally between stages of the PBP1b activity cycle, with LpoB additionally inducing arrangements that would prime or favour active conformations. The interface between the UB2H domain and the TPase and GTase domains has been explored previously using point mutations of hydrogen bonding networks observed in the MoeA-liganded structures of PBP1b^12^. Here, we analyse the differential hydrogen bonding of the apo form in the context of these known amino acid substitution effects.

**Fig. 3:**
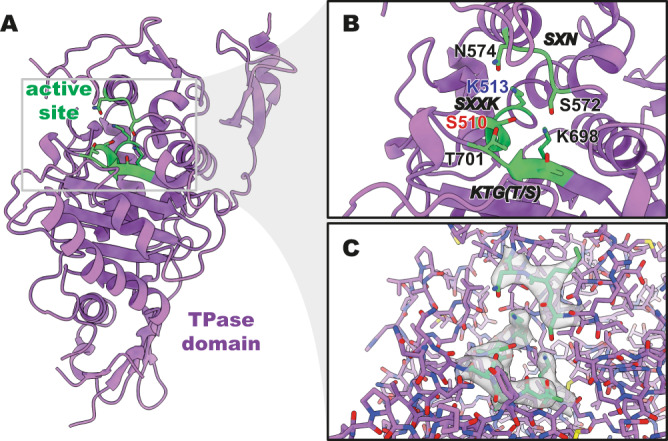
Transpeptidase domain of apo PBP1b. **A** An overview of the structure transpeptidase domain (TPase—purple). The *SXXK*, *SXN* and *KTG(T/S)* motifs of the active site are in green, with the active site region boxed in grey. **B** A close-up of the active site of the TPase domain of PBP1b. The motifs, as in (**A**), are in green. The *SXXK* motif contains both the serine nucleophile (S510—red) and the general base lysine (K513—blue). The *SXN* motif is involved in the protonation of the nitrogen leaving group during acylation, while the *KTG(T/S)* motif forms the oxyanion hole and is involved in substrate binding. **C** The active site view, as in (**B**), in stick representation along with the transparent cryoEM map of PBP1b around the active site residues. Despite the modest resolution for the overall structure (3.28 Å), the density of the TPase domain-active site is above the mean (at ~3.1–3.2 Å) due to its recessed location and the overall higher local resolution observed for the TPase domain (Supplementary Fig. 3).

Between the apo and MoeA-liganded structures of PBP1b, we see a number of conserved residues playing analogous roles in both (Fig. 3B, C). Most notable are conserved hydrogen bonds at the interface of the UB2H and TPase domains (Fig. 3B, C), including those between the side chain guanidino group of R109 and carboxylic acid of D443 (R109-D443) and the interaction between the backbone amide nitrogen of R109 and backbone carbonyl oxygen of F422 (R109-F442). Moreover, we see a role for S444 in both the apo and liganded state, with the side chain hydroxyl of S444 forming a hydrogen bond interaction with the backbone carbonyl oxygen of A130 (A130-S444) in the apo PBP1b structure, while forming an interaction with the guanidino group of R200 (R200-S444) in the liganded PBP1b structure^3^. Each of the states has one additional hydrogen bond interaction unique to that state; in the apo PBP1b structure formed between the carboxylic acid group of E123 and the hydroxyl of T751 (E123-T751) and in the MoeA-liganded structure^3^, between the ε-amino group of K126 and the backbone carbonyl oxygen of N749 (K126-N749). These non-conserved interactions are localized in close proximity due to the minimal domain rearrangement between the UB2H and TPase domains.

The interactions between the UB2H and GTase domains, on the other hand, are more differentiated, not surprising given the larger observed interdomain movements between apo and MoeA-liganded structures of PBP1b (Fig. 3B, C). Only R134 is observed to play a hydrogen bonding role in both states. In the apo form, R134 adopts an extended rotamer conformation, with its guanidino group forming hydrogen bond interactions to both the carboxylic acid group of D345 and the backbone carbonyl of P398 (R134-D345 and R134-P398). In the MoeA-liganded state^3^, the guanidino group of R134 is rotated inward, toward the UB2H domain’s centre of mass, forming hydrogen bonding and electrostatic interactions with the carboxylic acid group of E341 (R134-E341). The latter also interacts with the guanidino group of the adjacent R157 (R157-E341). Two additional interactions observed exclusively in the apo PBP1b structure include a hydrogen bond between the hydroxyl group of T140 with the guanidino group of R397 (T140-R397) and a second ion pair interaction from the latter moiety with the carboxylic acid side chain of E144 (E144-R397).

We observe that the interfaces between the TPase, UB2H and GTase domains are complex and dynamic amongst known conformations of PBP1b. We have now resolved the structure of PBP1b in one additional state, the apo form, the starting point of the catalytic cycle of processive polymerization and transpeptidation, with likely additional states to be observed in order to fully unravel the conformational plasticity of PBP1b needed for its multivalent catalytic activities.

### TPase domain of apo *E. coli* PBP1b

The TPase domains of both class A and B PBPs are longstanding drug targets, through their inhibition by β-lactam antibiotics. Despite the relative ease with which one can crystallize monofunctional class B PBPs (as demarcated by the abundance of class B PBP structures from a variety of species deposited in the PDB), class A PBPs have been rather recalcitrant to crystallization, with class A PBPs (or TPase containing portions of class A PBPs) only having been solved from five distinct species (*Staphylococcus aureus* PBP2^5,6^, *E. coli* PBP1b^3,7,8^, *Acinetobacter baumannii* PBP1a^13^, *Listeria monocytogenes* PBP1a^14^ and *Haemophilus influenzae* PBP1a—Supplementary Table 2). Here, we show the potential for the structural determination of further class A PBPs using cryoEM and provide a glimpse at the potential for the use of cryoEM structure-guided drug design for class A PBPs.

In our apo structure of *E. coli* PBP1b, we see clear density for the majority of the TPase domain. With the exception of four unresolved residues on the membrane distal loop (543–546), the TPase domain is as modelled in crystal structures (Fig. 3A) and has a backbone root-mean-square deviation (RMSD) of 0.6 Å across 364 residues in comparison to the acyl-CENTA-liganded structure in the detergent (PDB 5HLD^3^). We note that this close overlap also supports that the SMA lipid particle (SMALP) detergent mimetic here has no conformational influence when compared to the liganded structure determined by X-ray crystallography in a detergent micelle. The local resolution for the TPase domain is seen to be higher than the overall resolution of 3.28 Å, with local resolution at ~3.1–3.3 Å (Supplementary Fig. 3). Due to the nature of cryoEM data processing, local resolution estimates usually radiate outward from a centre of mass, with the peripheral portions of a cryoEM map usually being estimated at lower than average resolutions. The peripheries of the TPase domain are seen to be estimated at ~3.2–3.3 Å resolution, although, due to the recessed nature of the TPase active site, resolution in the active site is estimated to be ~3.1–3.2 Å. While resolution in the 3 Å range is typically not the target for structure-guided drug design, recent advances in cryoEM model building^15^ and cryoEM ligand fitting^16^ allow confident assignment of ligand poses in low 3 Å cryoEM density and this can be quite indicative of downstream higher-resolution pursuits. In the apo structure, we observe all of the expected features of a TPase domain, with clear density for all of the residues that make up the canonical *SXXK*, *SXN*, and *KTG(T/S)* motifs (Fig. 3B, C).

### GTase domain of apo *E. coli* PBP1b

**Fig. 4:**
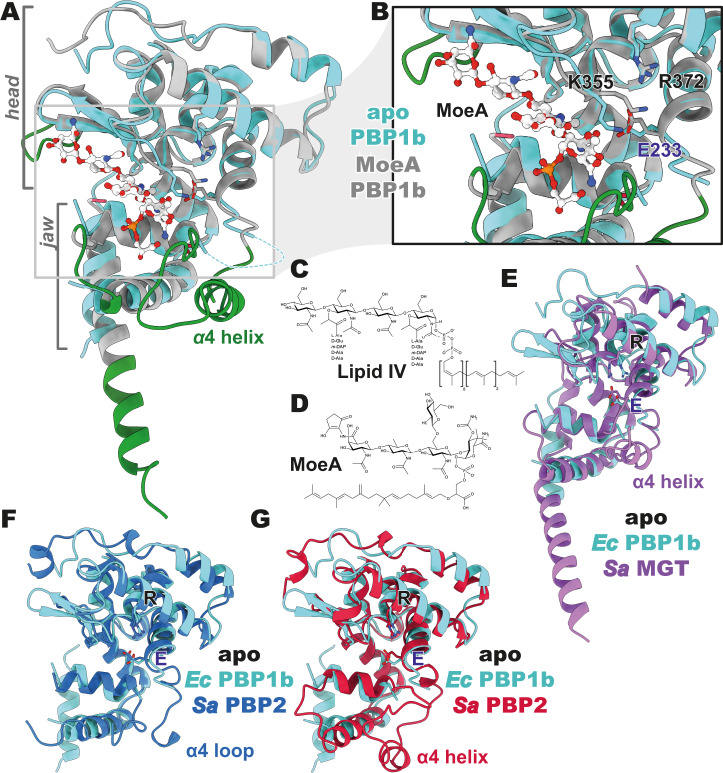
Glycosyltransferase domain of apo PBP1b. **A** An overlay of the GTase domains of the apo structure of *E. coli* PBP1b- (cyan) and a moenomycin A- (MoeA) bound crystal structure (grey—PDB 5HLB^3^). Regions resolved in the MoeA-bound crystal structure are coloured in green. Notably, a lack of stabilization by MoeA leads to the unresolved nature of the α4 helix loop in the apo PBP1b structure. Both the head and jaw regions are labelled in grey. **B** A view of the active site of the GTase domains, as in (**A**), highlighting the relatively conserved nature of the active site. The catalytic general base, E233, is labelled in blue font, while the K355 and R372, which modulate the p*K*_a_ of the E233 carboxylate side chain, are labelled in black. **C**, **D** A comparison of lipid IV, a substrate of the GTase domain (**C**) and moenomycin A (MoeA), a lipid IV substrate analogue inhibitor (**D**). **E**–**G** A comparison of the apo *E. coli* PBP1b GTase domain structure (cyan) to the three published crystal structures of apo GTase domains. Conserved glutamate general base and p*K*_a_-modulating arginine are shown and labelled in blue and black, respectively. **E** Apo *E. coli* PBP1b GTase domain compared to the apo structure of the monofunctional GTase, MGT, of *S. aureus* (purple—PDB 3VMQ^17^). **F** Apo *E. coli* PBP1b GTase domain compared to the first apo structure of the bifunctional PBP, PBP2, of *S. aureus* (blue—PDB 2OLU^6^). **G** Apo *E. coli* PBP1b GTase domain compared to the second apo structure of the bifunctional PBP, PBP2, of *S. aureus* (red—PDB 3DWK^5^).

For the GTase domain of apo *E. coli* PBP1b, we see that the overall estimated local resolution is lower than that of the TPase domain, at ~3.4–3.7 Å (Supplementary Fig. 3). Lower resolution in the GTase domain of class A PBPs is expected and observed in crystal structures of class A PBPs, due to increased dynamic motion of these domains, a hallmark of their processive polymerizing activity^3,6,7^. The modelled regions of this domain are similar to what was modelled previously for the partially disordered MoeA-liganded GTase domain of PBP1b (PDB 3VMA^7^, 5HLD^3^, etc.), with the exception of the lone structure of MoeA-liganded PBP1b where the α4 helix loop is stabilized and modelled (PDB 5HLB^3^). If we use the more complete 5HLB as a model for comparison between our apo and liganded PBP1b GTase domains (Fig. 4A), we observe that the overall fold and domain architecture of the GTase domain is conserved. We see that both the globular head and jaw region are in the same place, with the only major differences (α4 helix loop aside) being in the short loop regions between secondary structure elements. Aligning the two, we see a backbone RMSD of 3.6 Å across all 138 residue pairs and, when pruning back the loop regions, we observe a backbone RMSD of 1.0 Å across 109 residue pairs.

The active site of the GTase domain is well conserved between the liganded and apo states (Fig. 4B), with both the E233 catalytic general base and R372, predicted to modulate the p*K*_a_ of E233^3^, observed in similar positions. K355, which additionally could act to modulate the p*K*_a_ of the catalytic E233^3^, is seen to protrude further into the active site cleft in the apo structure. In the MoeA-liganded structure^3^, this residue is rotated back toward R372. This could be due to the location of the bound MoeA, which would otherwise form unfavourable steric or charged interactions with K355 (Fig. 4B). It is possible that this rotation is what would occur upon binding of the natural substrate, lipid II(+2*n*), of which MoeA is an analogue (Fig. 4C, D), although differences in MoeA versus lipid IV could potentially allow for a more favourable interaction between K355 and the bound ligand in the case of the natural substrate.

Comparing the apo GTase domain of *E. coli* PBP1b to that of other PG GTase domains that have been determined, we notice that the majority of the GTase domain-containing structures are either MoeA bound or have completely disordered (and unmodelled) jaw regions of the GTase domain (Supplementary Fig. 4 and Supplementary Table 2). Because of this, there are only three apo structures to compare; a monofunctional GTase, MGT (3VMQ^17^) at 2.52 Å resolution, and two structures of PBP2 at 2.90 and 3.10 Å resolution, the class A PBP (2OLU^6^, 3DWK^5^), all from *S. aureus*. Similar to what was observed amongst *E. coli* PBP1b GTase structures, we see high structural conservation on the level of secondary structure when comparing apo PBP1b GTase domain to the apo GTase domains of MGT and PBP2 (Fig. 4E–G). When divergent loops are pruned, we see backbone RMSDs of 1.1 Å across 82 residues for MGT (3VMQ), and 1.0 Å across 94 and 93 residues for both structures of PBP2 (2OLU and 3DWK, respectively).

Amongst the observed structures of GTase domains, the main structural differences come down to the position and stabilization of the α4 helix loop. In the case of most structures of GTase domains where the entirety of the α4 helix loop could be modelled, there are contacts with adjacent molecules in the asymmetric unit. Two such examples are the structure of *S. aureus* PBP2 (apo—3DWK^5^) and MGT (MoeA liganded—6FTB^18^). Despite the potential artifactual positioning/ordering by crystal contacts of this otherwise dynamic helix, these structures have allowed for insight into the potential interfaces between ligands and the α4 helix loop. In the case of some of the MoeA-liganded structures, such as the *E. coli* PBP1b structure 5HLB, we see stabilization that could be more indicative of a natural state, as the stabilization of the α4 helix loop is in part driven by interactions with the natural substrate analogue MoeA. From our cryoEM and detergent-free structure of *E. coli* PBP1b in an apo state, we propose that this loop is forming a spectrum of conformations in the absence of substrate or inhibitor, as density for this region is not observed. Indeed, this ability to rapidly unfold in the absence of donor substrate is a major premise of earlier models of processive polymerization, providing a path for new product to slot into the donor site^8,10,18^.

In this work, we have shown the structure of the 83 kDa class A PBP, PBP1b, from *E. coli*. This was solved in a detergent-free system, using cryoEM. In comparison to MoeA-liganded structures of PBP1b, the apo form is seen to exhibit a distinct conformation. We observe the dynamic domain interface rearrangements between the TPase, UB2H, and GTase domains of PBP1b. On a domain level, we note that there is clear density for the TPase domain, and we propose that our methodology could be repurposed to play a key role in the structure-guided drug design for this notoriously difficult to crystalize class of proteins. In addition, from the apo structure of *E. coli* PBP1b, we are able to compare and contrast with both liganded forms of *E. coli* PBP1b, as well as apo forms of PG GTases from other species. We hope that the techniques described in this work can stimulate the pursuit of further cryoEM structures of class A PBPs from additional species, such as those from ESKAPE pathogens^19^ and WHO priority pathogens^20^ with no current structural information and little sequence identity, typically in the range 30–40%. Furthermore, we hope that this cryoEM methodology will allow for the capture of structural information regarding the native acceptor and donor substrates, which have largely eluded crystallization in both class A and class B PBPs. Structural insight into these acceptor and donor substrates will allow for further appreciation of recent work to unravel these mechanisms through the use of continuous assays^21^ and allow for the development of novel antibacterial compounds that target GTase activity. We believe that the work presented in this manuscript will revitalize the study of class A PBPs and that this and subsequent work will be of great assistance as we attempt to ward off a post-antibiotic era.

## Methods

### Cloning and protein expression

*Escherichia coli* PBP1b (residues 58–804) and LpoB (resides 78–213) were cloned into the expression vector pET41b with a thrombin-cleavable, C-terminal His_8_-tag^8^. Expression constructs were transformed into *E. coli* C41 (DE3) for expression. Cells were cultured in ZYP-5052 autoinduction media for 4 h at 37 °C followed by overnight protein expression at 25 °C. Cells were pelleted and stored at −80 °C until required.

### Protein purification

For purification of *E. coli* PBP1b^22^, cell pellets were resuspended in lysis buffer (20 mM HEPES, pH 8.0, 300 mM NaCl, 10% glycerol) and lysed by processing twice with a homogenizer (15 kPa; Avestin). Cellular debris was pelleted by centrifugation at 10,000 × *g* for 30 min. The resultant supernatant was centrifuged at 125,000 × *g* for 1 h to pellet membranes. The membranes were solubilised in Buffer A (20 mM HEPES, pH 8.0, 300 mM NaCl) with 1% (w/v) DDM for 1 h at 4 °C and loaded onto 5 mL Ni-NTA Superflow resin (Qiagen), washed with 75 mM imidazole in Buffer A with 0.016% DDM, and the protein was eluted with 300 mM imidazole. One unit of thrombin was added per mg of protein to remove the N-terminal His-tag overnight at 4 °C. Samples were exchanged into SMA (SMALP 30010P, PolyScience) by SEC with a Superdex 200 column (GE Lifesciences) equilibrated in Buffer A with no DDM. Fractions containing pure PBP1b were pooled. LpoB was purified as described previously^23^. To assay lipoprotein activator binding, lipoprotein activator was added to the sample via glycerol gradient centrifugation on a 5–25% gradient overnight at ~30,000 × *g*.

### Cryo-electron microscopy

Aliquots of 3 μL of SMA-PBP1b and a 4-fold molar excess of LpoB were applied to glow-discharged Quantifoil® (1.2/1.3) grids. The grids were blotted for 3 s at 100% humidity with an offset of 3 and plunge frozen into liquid ethane using a Vitrobot Mark IV (Thermo Fisher). Grids were imaged on a 300 keV Titan Krios cryo-electron microscope (Thermo Fisher) equipped with a K3 camera (Gatan). Movies were collected at a calibrated magnification of ×105,000, corresponding to a 0.844 Å per physical pixel. The dose was set to a total of 60 electrons/Å^2^ over an exposure of 50 frames. Automated data collection was carried out using SerialEM with a nominal defocus range set from −0.8 to −2.0 μM. A total of 12,788 movies were collected over 72 h.

### Image processing

The 12,788 movies were motion-corrected using MotionCor2^24^ and micrographs were binned to 0.844 Å/pixel. The contrast transfer functions (CTFs) of the flattened micrographs were determined using CTFFIND4.1^25^. The micrographs were denoised using JANNI^26,27^, and subsequently, 5,279,209 particles were picked using crYOLO^26^. Particles were additionally binned to 3.376 Å/pixel and reference-free 2D classification was performed in cryoSPARC^28^, leaving 4,554,764 particles after obvious junk and ice was removed. A subset consisting of the first 4886 micrographs of collection was processed in a similar way. Four ab initio models were generated and subsequently heterogeneously refined in cryoSPARC^28^. The most reasonable class was subjected to another round of ab initio modelling and heterogeneous refinement. The most reasonable class and a junk class were used in three rounds of iterative heterogenous refinement to reduce the particle stack to 1,929,725 particles, which were limited by the binned pixel size. The particles were unbinned to a pixel size of 1.688 Å and the iterative refinement was continued for six more iterations, resulting in a stack of 1,043,248 particles. At this point, additional 8-class ab initio modelling was performed, followed by an 8-class heterogenous refinement. The best class resulted in 462,997 particles, which were subsequently unbinned to 0.844 Å/pixel. These particles were refined using cryoSPARC local refinement and non-uniform refinement^29^, with the fulcrum set at the centre of mass. This volume was then reconstructed in RELION 3.1^30^ and Bayesian polishing was performed. The polished particle stack was then locally refined again in cryoSPARC^28^, and again reconstructed in RELION 3.1^30^. Particle subtraction was performed to remove SMA, lipid, and dimeric PBP1b density. The subtracted particles were then once again locally refined in cryoSPARC^28^, and globally and locally CTF refined in RELION 3.1^30^. Final refinement was performed in RELION 3.1^30^ to a resolution of 3.28 Å. The resolution was determined at 0.143 criterion using the Fourier shell correlation gold-standard refinement procedure. The final map was sharpened using LocScale^31^. Throughout the processing pipeline, no density for LpoB was observed in any classes.

### Model building and refinement

Our previous structure of liganded PBP1b (PDB 5HLD) was split into TPase-UB2H and GTase parts and docked into the map using UCSF Chimera^11^. The resultant model was then refined using Phenix real-space refine^32^, ISOLDE^15^ and manual building in Coot^33^. The final model has a good fit to the map (EMRinger^34^ score 3.35) and good statistics (MolProbity^35^ score 1.86, Ramachandran favoured 93.98% and outliers 0.15%).

### Reporting summary

Further information on research design is available in the Nature Research Reporting Summary linked to this article.

## Supplementary information

Supplementary Information

Reporting Summary

## Data Availability

Data supporting the findings of this manuscript are available from the corresponding author upon reasonable request. A Reporting summary for this article is available as a Supplementary information file. CryoEM maps and atomic coordinates for *E. coli* PBP have been deposited in the EMDB (EMD-23482) and PDB (7LQ6) respectively. Source data are provided with this paper.

## Source data

Source Data

## References

[1] Caveney NA, Li FK, Strynadka NC (2018). Enzyme structures of the bacterial peptidoglycan and wall teichoic acid biogenesis pathways. Curr. Opin. Struct. Biol..

[2] Sobhanifar S, King DT, Strynadka NCJ (2013). Fortifying the wall: synthesis, regulation and degradation of bacterial peptidoglycan. Curr. Opin. Struct. Biol..

[3] King DT, Wasney GA, Nosella M, Fong A, Strynadka NCJ (2017). Structural insights into inhibition of Escherichia coli penicillin-binding protein 1B. J. Biol. Chem..

[4] King DT, Sobhanifar S, Strynadka NCJ (2016). One ring to rule them all: current trends in combating bacterial resistance to the β-lactams. Protein Sci..

[5] Lovering AL, De Castro L, Strynadka NCJ (2008). Identification of dynamic structural motifs involved in peptidoglycan glycosyltransfer. J. Mol. Biol..

[6] Lovering AL, De Castro LH, Lim D, Strynadka NCJJ (2007). Structural insight into the transglycosylation step of bacterial cell-wall biosynthesis. Science.

[7] Sung M (2009). Crystal structure of the membrane-bound bifunctional transglycosylase PBP1b from *Escherichia coli*. Proc. Natl Acad. Sci. USA.

[8] King AM (2016). Structural and kinetic characterization of diazabicyclooctanes as dual inhibitors of both serine-β-lactamases and penicillin-binding proteins. ACS Chem. Biol..

[9] Sun C, Gennis RB (2019). Single-particle cryo-EM studies of transmembrane proteins in SMA copolymer nanodiscs. Chem. Phys. Lipids.

[10] Egan AJF (2014). Outer-membrane lipoprotein LpoB spans the periplasm to stimulate the peptidoglycan synthase PBP1B. Proc. Natl Acad. Sci. USA.

[11] Pettersen EF (2004). UCSF Chimera—a visualization system for exploratory research and analysis. J. Comput. Chem..

[12] Egan AJF (2018). Induced conformational changes activate the peptidoglycan synthase PBP1B. Mol. Microbiol..

[13] Han S (2011). Distinctive attributes of β-lactam target proteins in *Acinetobacter baumannii* relevant to development of new antibiotics. J. Am. Chem. Soc..

[14] Jeong JH (2013). Crystal structures of bifunctional penicillin-binding protein 4 from listeria monocytogenes. Antimicrob. Agents Chemother..

[15] Croll TI (2018). ISOLDE: a physically realistic environment for model building into low-resolution electron-density maps. Acta Crystallogr. D.

[16] Robertson MJ, van Zundert GCP, Borrelli K, Skiniotis G (2020). GemSpot: a pipeline for robust modeling of ligands into cryo-EM Maps. Structure.

[17] Huang C-Y (2012). Crystal structure of *Staphylococcus aureus* transglycosylase in complex with a lipid II analog and elucidation of peptidoglycan synthesis mechanism. Proc. Natl Acad. Sci. USA.

[18] Punekar AS (2018). The role of the jaw subdomain of peptidoglycan glycosyltransferases for lipid II polymerization. Cell Surf..

[19] Boucher HW (2009). Bad bugs, no drugs: no ESKAPE! An update from the Infectious Diseases Society of America. Clin. Infect. Dis..

[20] WHO. *Global Priority List of Antibiotic-resistant Batceria to Guide Research, Discovery, and Development of New Antibiotics*, 7pp (WHO, 2017).

[21] Catherwood AC (2020). Substrate and stereochemical control of peptidoglycan cross-linking by transpeptidation by *Escherichia coli* PBP1B. J. Am. Chem. Soc..

[22] Caveney NA (2019). Structural insight into YcbB-mediated beta-lactam resistance in *Escherichia coli*. Nat. Commun..

[23] King DT, Lameignere E, Strynadka NCJ (2014). Structural insights into the lipoprotein outer membrane regulator of penicillin-binding protein 1B. J. Biol. Chem..

[24] Zheng SQ (2017). MotionCor2: anisotropic correction of beam-induced motion for improved cryo-electron microscopy. Nat. Methods.

[25] Rohou A, Grigorieff N (2015). CTFFIND4: fast and accurate defocus estimation from electron micrographs. J. Struct. Biol..

[26] Wagner T (2019). SPHIRE-crYOLO is a fast and accurate fully automated particle picker for cryo-EM. Commun. Biol..

[27] Lehtinen, J. et al. Noise2Noise: learning image restoration without clean data. In *35th International Conference* on *Machine Learning ICML 2018*, Vol. 7, 4620–4631 (2018).

[28] Punjani A, Rubinstein JL, Fleet DJ, Brubaker MA (2017). CryoSPARC: algorithms for rapid unsupervised cryo-EM structure determination. Nat. Methods.

[29] Punjani A, Zhang H, Fleet DJ (2020). Non-uniform refinement: adaptive regularization improves single-particle cryo-EM reconstruction. Nat. Methods.

[30] Zivanov J (2018). New tools for automated high-resolution cryo-EM structure determination in RELION-3. Elife.

[31] Jakobi AJ, Wilmanns M, Sachse C (2017). Model-based local density sharpening of cryo-EM maps. Elife.

[32] Adams PD (2010). PHENIX: a comprehensive Python-based system for macromolecular structure solution. Acta Crystallogr. D.

[33] Emsley P, Cowtan K (2004). Coot: model-building tools for molecular graphics. Acta Crystallogr. D.

[34] Barad BA (2015). EMRinger: side chain-directed model and map validation for 3D cryo-electron microscopy. Nat. Methods.

[35] Chen VB (2010). MolProbity: all-atom structure validation for macromolecular crystallography. Acta Crystallogr. D.

