# Supplementary material for: CryoEM structure of the antibacterial target PBP1b at 3.3 Å resolution
Source: Nat Commun. 2021 May 13;12:2775. doi: 10.1038/s41467-021-23063-6 (PMC8119973; doi:10.1038/s41467-021-23063-6)
Supplement: Supplementary file 1 — Supplementary Information [file 41467_2021_23063_MOESM1_ESM.pdf]

CryoEM structure of the antibacterial target PBP1b at 3.3 Å resolution

Supplementary Information

Nathanael A Caveney[1,2], Sean D Workman[1,2], Rui Yan[3], Claire E Atkinson[1,2,4], Zhiheng Yu[3], Natalie C J Strynadka[1,2,4]*

[1] Department of Biochemistry and Molecular Biology, University of British Columbia, Vancouver, British Columbia, Canada

[2] The Centre for Blood Research, University of British Columbia, Vancouver, British Columbia, Canada

[3] CryoEM Shared Resources, Janelia Research Campus, Howard Hughes Medical Institute, Ashburn, VA, USA

[4] HRMEM Facility, University of British Columbia, Vancouver, British Columbia, Canada

* Correspondence: ncjs@mail.ubc.ca

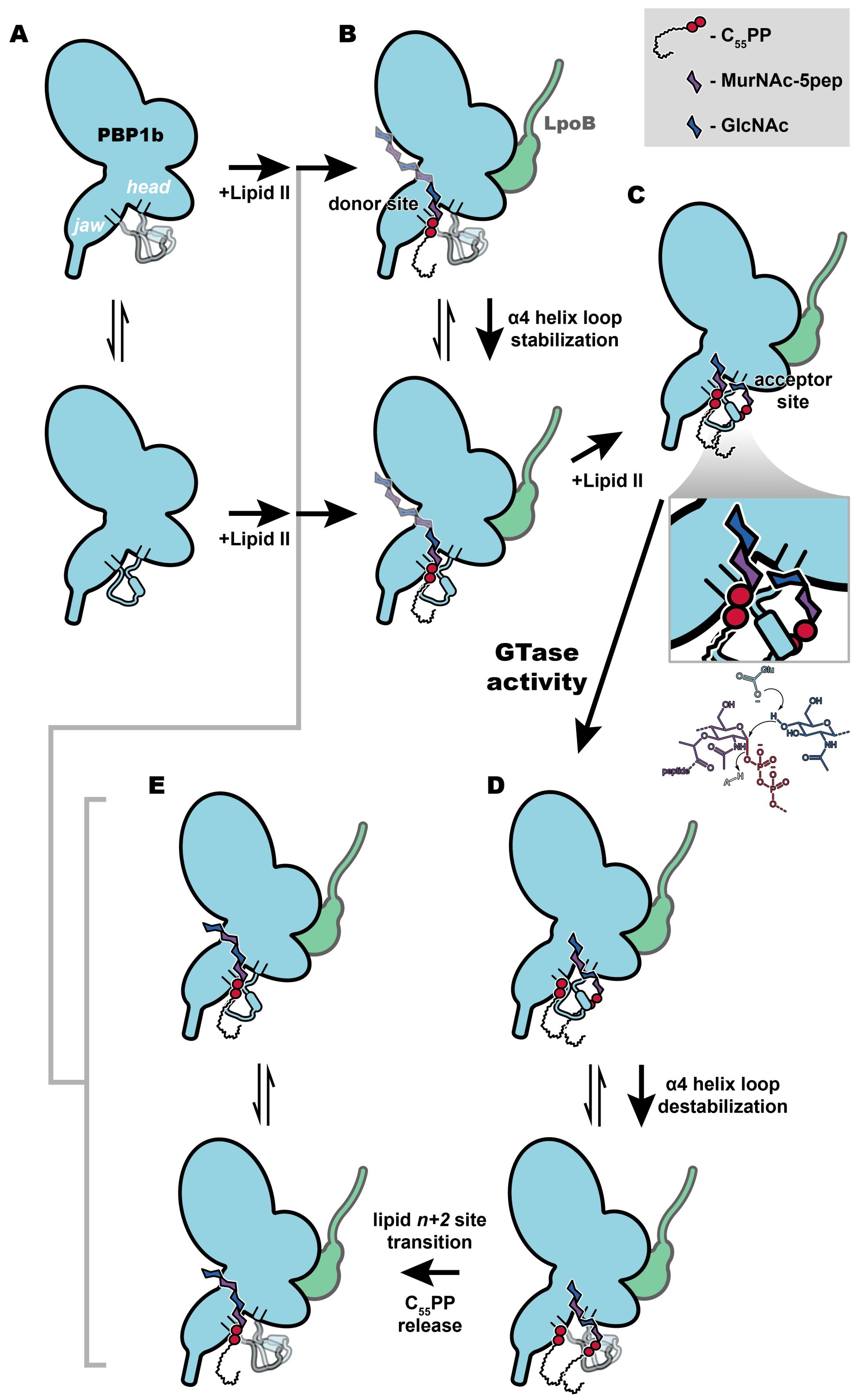

A
PBP1b
head
jaw
+Lipid II
B
LpoB
donor site
- C_{55}PP
- MurNAc-5pep
- GlcNAc
C
acceptor site
α4 helix loop stabilization
+Lipid II
+Lipid II
GTase activity
Glu
OH
HO
NH
peptide
D
E
α4 helix loop destabilization
lipid *n*+2 site transition
C_{55}PP release

Supplementary Figure 1. Schematic of proposed PG polymerization cycle of PBP1b. (**A**) The equilibrium state between apo PBP1b with and without a stabilized α4 helix loop. The destabilized α4 helix loop is as seen in the apo *E. coli* PBP1b structure reported in this work. (**B**) with the addition of lipid II, we see the formation of the initial lipid II-PBP1b complex, with the initial lipid II entering the donor site of PBP1b. This step is in equilibria between a stabilized and destabilized α4 helix loop. The ordered and disordered states are similar to what has been observed in MoeA bound structures of *E. coli* PBP1b (ordered – PDB 5HLB [1], disordered. – PDB 3VMA [2], 5HLD [1], etc.). (**C**) The ordered α4 helix loop state of the equilibrium in **B** provides a second site for the additional docking of a second lipid II. This lipid II is seen to sit in the acceptor site, as lipid II analogue was seen to do in the structure of *S. aureus* MGT (PDB 3VMT [3]), while the donor site lipid II is as in **B**. The state of PBP1b in **C** is primed for glycosyltransferase activity. (**D**) Glycosyltransferase activity occurs, resulting in the transfer of the donor disaccharide from lipid II (or, in subsequent cycles, the growing PG strand) to the acceptor lipid II. This is in equilibrium with a state with a destabilized α4 helix loop, to allow for subsequent lipid transition. (**E**) Lipid transition occurs, with the newly formed lipid IV (or, in subsequent cycles, lipid IV+*2n*) transitioning to the donor site, kicking out the released C_{55}PP carrier lipid. After transition, this state can once again form an equilibrium state both with and without a stabilized α4 helix loop and is equivalent to the state depicted in (**B**), yet with an additional MurNAc-pentapeptide – GlcNAc extension (+*2*). The cycle can then repeat to add additional disaccharide units to the growing peptidoglycan strand.

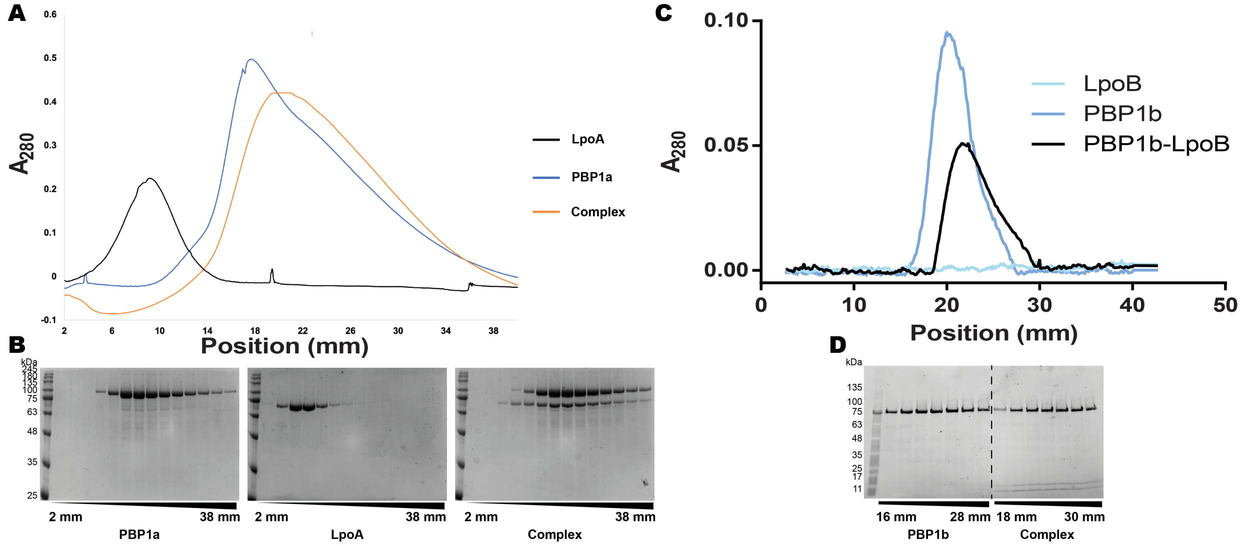

Supplementary Figure 2. SMA Solubilization of *E. coli* PBP1a and PBP1b. Assembly of PBP1-SMALP/LpoA PBP1-SMALP/LpoB complex using density gradient ultracentrifugation. **(A)** Overlaid fractionation chromatograms of LpoA (black), PBP1a-SMALP (blue), and PBP1a-SMALP/LpoA (orange) from separate 5-25% glycerol gradients. The shifted elution peak for PBP1a-SMALP compared to the PBP1a-SMALP/LpoA complex reflects successful complex assembly. This is confirmed by the shift in elution of LpoA observed in the SDS-PAGE gels in **(B)**. The first 2 mm of plunger movement are excluded as they only contained air and led to large spikes in the background UV absorbance. **(B)** SDS-PAGE gels corresponding to the 5-25% glycerol gradient fractionations above. **(C)** Overlaid fractionation chromatograms of LpoB (light blue), PBP1b-SMALP (black), and PBP1b-SMALP/LpoB (darker blue) from separate 5-25% glycerol gradients. The shifted elution peak for PBP1b-SMALP compared to the PBP1b-SMALP/LpoB complex reflects successful complex assembly. This is confirmed by SDS-PAGE gels in **(D)**. The first 2 mm of plunger movement are excluded as they only contained air and led to large spikes in the background UV absorbance. **(D)** SDS-PAGE gels corresponding to the peak fractions from the 5-25% glycerol gradient fractionations above. The glycerol gradients shown in this figure were repeated at a minimum 3 times over the course of cryoEM sample optimization, with similar results each repetition.

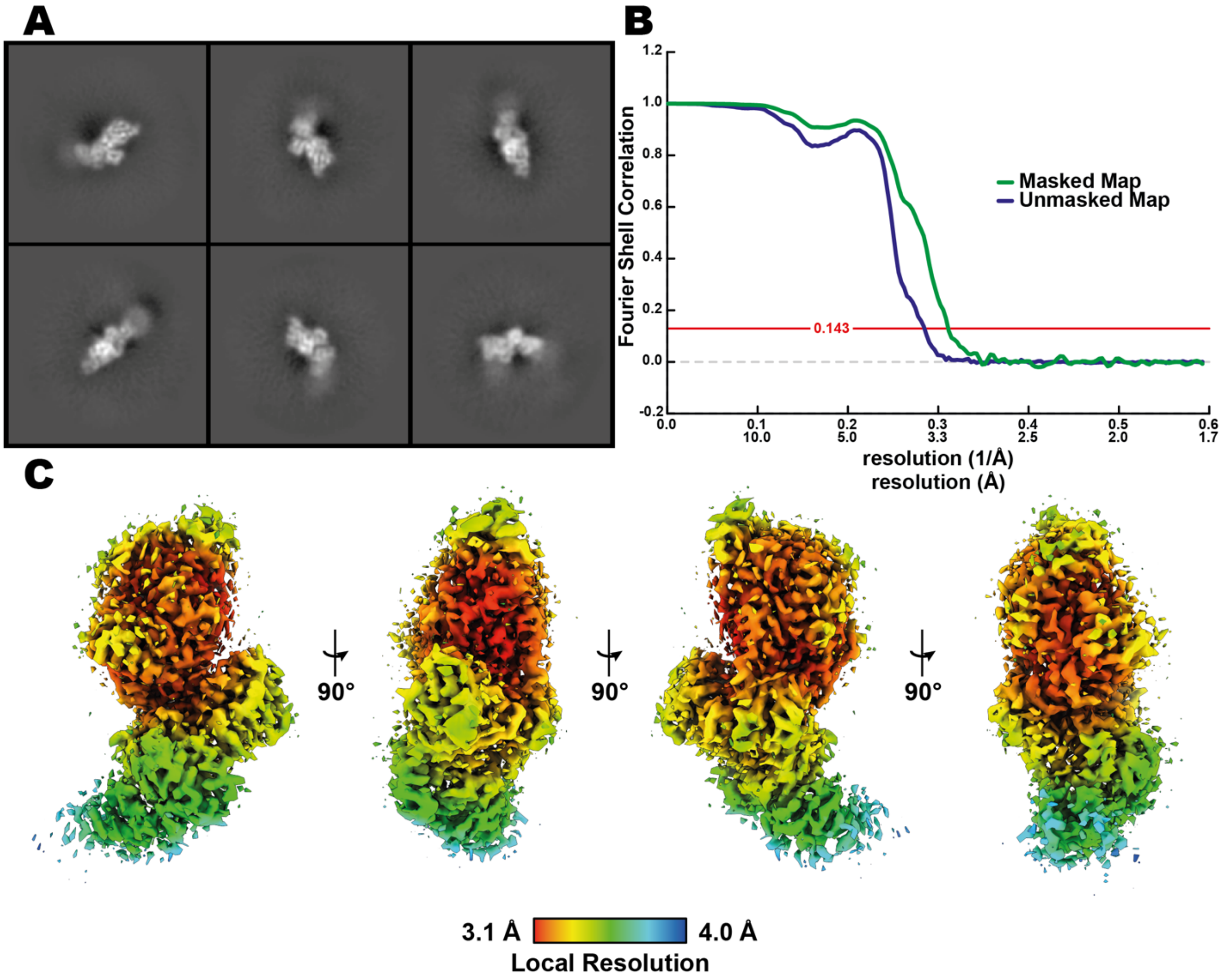

Supplementary Figure 3. 2D averages, Fourier shell correlation, and local resolution estimation for apo PBP1b. (**A**) Example reference-free 2D class averages of apo PBP1b. (**B**) FSC curve of the apo PBP1b reconstruction using gold-standard refinement calculated from unmasked and masked half maps. (**C**) Estimated local resolution, calculated by RELION 3.1 [4]. Resolution is coloured in rainbow from high (red – 3.1 Å) to low (blue – 4.0 Å). The TPase domain is highest resolution, at ~3.1-3.3 Å resolution, while the UB2H is at ~3.2-3.4 Å resolution and followed by the GTase domain at ~3.4-3.7 Å resolution.

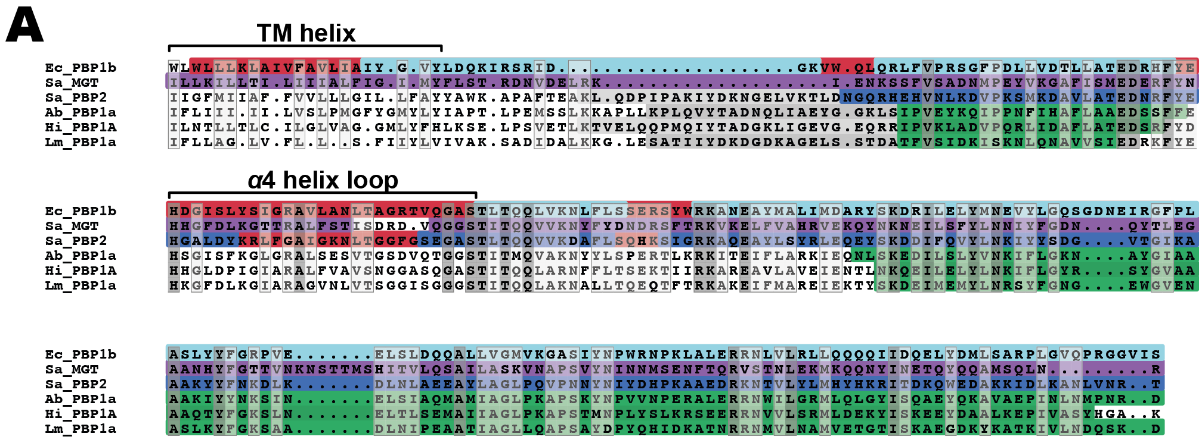

Supplementary Figure 4. GTase domain sequence alignments. (**A**) Protein sequence alignments for the 6 species with solved apo GTase domain structures. Elements of the sequence which were structured in the model are highlighted in colour. Colouring is as in Figure 4, with the addition of green for structures not in Figure F. The grey at the start of the highlighted regions for *S. aureus* PBP2 and *A. baumannii*, *H. influenzae*, and *L. monocytogenes* PBP1a are structured, but not part of the GTase domain. The extensive UB2H domain of PBP1b has been omitted from this alignment. Of note, only *E. coli* PBP1b and *S. aureus* MGT were solved with TM present. *S. aureus* PBP2 was solved without the TM present in the construct, and density is seen for the jaw region of the GTase domain. *A. baumannii*, *H. influenzae*, and *L. monocytogenes* PBP1a were solved without the TM present in the construct, and no density is seen for the jaw region.

Supplementary Table 1. CryoEM data collection, refinement, and validation statistics.

	apo PBP1b (PDB/EMDB 7LQ6/EMD-23482)
**Data collection and processing**	
Magnification	105,000
Voltage (keV)	300
Electron exposure ($e^-/Å^2$)	60
Defocus range (μm)	-0.8 to -2.0
Pixel size (Å)	0.844
Symmetry imposed	C1
Initial particle images	5,279,209
Final particle images	462,997
Map resolution FSC threshold (Å)	0.143
Map resolution (Å)	3.28
**Refinement**	
Initial model used (PDB)	5HLD
Model resolution FSC threshold (Å)	0.143
Model resolution (Å)	3.28
Map sharpening *B*-factor ($Å^2$)	150
Model Composition	
Non-hydrogen atoms	5267
Protein residues	672
Ligands	-
B-factors ($Å^2$)	
Protein	57.66
Ligand	-
R.m.s. deviations	
Bond lengths (Å)	0.005
Bond angles (°)	0.749
Validation	
MolProbity score	1.99
Clashscore	9.26
EMringer score	3.34
Rotamer outliers (%)	1.41
Ramachandran plot	
Favoured (%)	94.39
Allowed (%)	5.45
Outliers (%)	0.15

Supplementary Table 2. Class A PBP and monofunctional GTase structures.

PBP ID	Species	Type	GTase ligand	notes
3VMQ	*S. aureus*	mono	apo	GTase resolved
3VMR	*S. aureus*	mono	MoeA	
3VMS	*S. aureus*	mono	NBD-LII	
3VMT	*S. aureus*	mono	LII analogue	liganded
6FTB	*S. aureus*	mono	MoeA	
3HZS	*S. aureus*	mono	MoeA	
3UDF	*A. baumannii*	bi	apo	
3UDI	*A. baumannii*	bi	apo	
3UDX	*A. baumannii*	bi	apo	
3UE0	*A. baumannii*	bi	apo	
3UE1	*A. baumannii*	bi	apo	
5U2G	*H. influenzae*	bi	apo	GTase domain jaw region not resolved
3ZG7	*L. monocytogenes*	bi	apo	
3ZG8	*L. monocytogenes*	bi	apo	
3ZG9	*L. monocytogenes*	bi	apo	
3ZGA	*L. monocytogenes*	bi	apo	
2OQO	*A. aeolicus*	cut-bi	HEPES/CHAPS	
3D3H	*A. aeolicus*	cut-bi	neryl-MoeA	
3NB6	*A. aeolicus*	cut-bi	neryl-MoeA (+ CH_3)	
3NB7	*A. aeolicus*	cut-bi	neryl-MoeA (- COO^-)	
3FWL	*E. coli*	bi	MoeA	
5FGZ	*E. coli*	bi	MoeA	
5HL9	*E. coli*	bi	MoeA	liganded
5HLA	*E. coli*	bi	MoeA	
5HLB	*E. coli*	bi	MoeA	
5HLD	*E. coli*	bi	MoeA	
3VMA	*E. coli*	bi	MoeA	
2OLV	*S. aureus*	bi	MoeA	
3DWK	*S. aureus*	bi	apo	GTase resolved
2OLU	*S. aureus*	bi	apo	

Supplementary References

1. King, D. T., Wasney, G. A., Nosella, M., Fong, A. & Strynadka, N. C. J. Structural Insights into Inhibition of Escherichia coli Penicillin-binding Protein 1B. *J. Biol. Chem.* **292**, 979–993 (2017).

2. Sung, M. *et al.* Crystal structure of the membrane-bound bifunctional transglycosylase PBP1b from Escherichia coli. *Proc. Natl. Acad. Sci. U. S. A.* **106**, 8824–8829 (2009).

3. Huang, C.-Y. *et al.* Crystal structure of Staphylococcus aureus transglycosylase in complex with a lipid II analog and elucidation of peptidoglycan synthesis mechanism. *Proc. Natl. Acad. Sci.* **109**, 6496–6501 (2012).

4. Zivanov, J. *et al.* New tools for automated high-resolution cryo-EM structure determination in RELION-3. *Elife* **7**, 1–22 (2018).
